# A sustainable approach for the degradation kinetics study and stability assay of the SARS-CoV-2 oral antiviral drug Molnupiravir

**DOI:** 10.1038/s41598-023-34537-6

**Published:** 2023-05-31

**Authors:** Fadwa H. Edrees, Mohammed E. Draz, Ahmed S. Saad, Sherif F. Hammad, Heba M. Mohamed

**Affiliations:** 1 Pharmaceutical Chemistry Department, Faculty of Pharmacy, Nahda University (NUB), Beni Suef, 62511 Egypt; 2 Department of Pharmaceutical Chemistry, Faculty of Pharmacy, Delta University for Science and Technology, Gamasa, Egypt; 3 Present Address: Analytical Chemistry Department, Faculty of Pharmacy, Cairo University, Kasr El-Aini St, Cairo, 11562 Egypt; 4 Medicinal Chemistry Department, PharmD Program, Egypt-Japan University of Science and Technology (E-JUST), New Borg El-Arab City, 21934 Alexandria Egypt; 5 Pharmaceutical Chemistry Department, Faculty of Pharmacy, Helwan University, Helwan, Egypt

**Keywords:** Mass spectrometry, Drug discovery

## Abstract

Molnupiravir (MPV) is the first direct-acting oral antiviral drug that effectively decreases nasopharyngeal infections with SARS-CoV-2 virus. The stability of MPV was tested by subjecting the drug to various stress conditions. The drug is liable to oxidative, acidic, and alkaline degradation and showed significant stability against thermal degradation. Mass spectrometry identified the degradation products and guided suggestion of the degradation patterns. Interestingly, while inspecting the UV-absorption spectra, we observed no absorbance at 270 nm for the products of the three degradation pathways (c.f. intact MPV). Direct spectrophotometry seemed a solution that perfectly fit the purpose of the stability assay method in our case. It avoids sophisticated instrumentation and complex mathematical data manipulation. The method determined MPV accurately (100.32% ± 1.62) and selectively (99.49% ± 1.63) within the linear range of 1.50 × 10^–5^–4.0 × 10^–4^ M and down to a detection limit of 0.48 × 10^–5^ M. The proposed method is simple and does not require any preliminary separation or derivatization steps. The procedure proved its validity as per the ICH recommendations. The specificity was assessed in the presence of up to 90% degradation products. The study evaluated the greenness profile of the proposed analytical procedure using the National Environmental Methods Index (NEMI), the Analytical Eco-Scale, and the Green Analytical Procedure Index (GAPI). The three metrics unanimously agreed that the developed procedure results in a greener profile than the reported method. The method investigated the degradation reactions' kinetics and evaluated the reaction order, rate constant, and half-life time for each degradation process.

## Introduction

Since 2019, humanity has been struggling due to the outbreak of COVID-19 caused by the SARS-Cov-2 virus. The symptoms range from fever, cough, shortness of breath, and fatigue in mild cases to life-threatening trouble breathing and persistent chest pain in severe cases. From the beginning of the crisis until April 2023, the WHO confirmed around 762 million COVID-19 cases and 6.89 million deaths, despite the administration of 13.3 billion vaccine doses^1^. The pandemic led to severe health, social and economic crises. Given the continuous mutation of the virus that led to SARS-CoV-2 and the emergence of different variants, thus it mandates holistic efforts for infection control and treatment plans, with particular emphasis on antiviral medications^2^.

**Figure 1:**
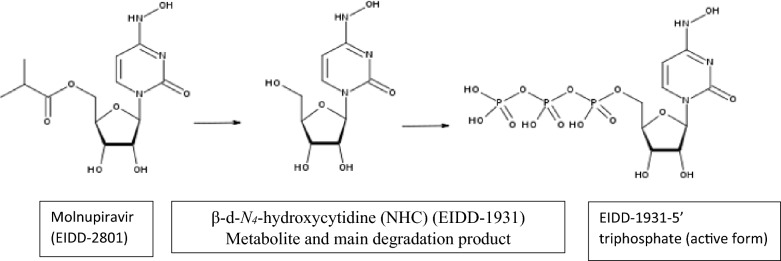
Chemical structure of molnupiravir, its active metabolites, and the hydrolysis degradation product.

Pharmacists, chemists, and physicians have been seeking promising solutions for effective diagnosis, control, treatment, and suppression of COVID-19. Molnupiravir (MPV), [(2R,3S,4R,5R)-3,4-dihydroxy-5-[4-(hydroxyamino)-2 oxopyrimidin-1-yl] oxolan-2-yl] methyl 2-methyl propanoate^3^, structure shown in (Fig. 1), is the antiviral prodrug of β-d-*N*_*4*_-hydroxycytidine (NHC)—a nucleoside derivative—which suppresses the replication of different RNA viruses^4^. MPV was approved as the first oral antiviral for COVID 19 by the Medicines and Healthcare products Regulatory Agency (MHRA) in the UK in November 2021^2^. By the end of December 2021, FDA released an emergency use authorization (EUA) for MPV for treatment of certain COVID-19 cases, mainly adults suffering from mild to moderate symptoms and those susceptible to turn to severe or hospitalized cases^5^. MPV exhibited a broad-spectrum antiviral efficiency against SARS-CoV-2, SARS-CoV, MERS-CoV, and bat-CoVs^4^. It exerts antiviral activity through the induction of several mutations in the viral RNA genome. MPV incorporates the NHC triphosphate rather than real uridine triphosphate or cytidine triphosphate by RNA-dependent RNA polymerase. In addition, the NHC triphosphate behaves like a chain terminator, hindering viral replication^6^. These frequent mutations cause an effect known as lethal mutagenesis or viral error catastrophe that makes the virus unable to survive^4,6^. MPV effectively and safely decreases the risk of hospitalization and death in patients with mild to moderate COVID-19^2^. It remarkably enhanced the functions of the lungs and reduced the virus titer^4^.

A validated LC/MS/MS method was reported to determine MPV and its metabolite in human plasma and saliva using acetonitrile in the mobile phase^7^. The primary active metabolite of MPV was also assayed in peripheral blood mononuclear cell lysates by LC/MS/MS^4^. Another study used the RP-HPLC method to determine MPV and its stability^8^. Although the reported chromatographic methods seem sensitive and selective, they do not consider the methodology's environmental impact, in addition to being sophisticated and requiring expensive instrumentation with trained labor. Accordingly, they cannot fit routine analysis in quality control laboratories. Recently, an electrochemical determination of MPV using a composite with cobalt (III) oxyhydroxide is theoretically calculated using mathematical model analysis^9^. There is a clear gap in the literature on studying the stability of MPV through the effect of various forced degradation conditions, proposing the degradation pattern, elucidating the degradation products, and determining the degradation kinetics, especially using a simple, environment-friendly spectrophotometric analysis. As commonly understood that spectrophotometry introduces easy, affordable, and reliable analytical solutions in quality control laboratories for routine drug analysis. Although the non-selective additive light absorption property of the matter seems to hinder the selective assay of mixtures, several spectrophotometric methods manipulated the absorption spectra to determine binary mixtures^10–12^, ternary mixtures^13^, and complex mixtures^14,15^, which in turn expanded the range for the spectrophotometric technique.

Since Molnipiravir is a recent discovered antiviral drug, thus it is very crucial to study the stability and the degradation patten of the drug molecule through the force degradation studies, this usually takes place under various stress conditions such as temperature, humidity, light, and mechanical stress^16,17^. The purposes of the forced degradation studies is to help identifyiung the potential degradation pathways and impurities that might be formed during the manufacturing, storage, and transportation of various drug products and to provide evidence on how the quality of a drug substance or drug product varies with time under the influence of various environmental conditions^18^. The outcomes of such studies can be used as a guidance for selecting the most appropriate formulation, packaging materials, and storage conditions to safeguard the stability and efficacy of drug products throughout their shelf-life^16^.

The main goals of our research are (1) to study the impact of forced degradation conditions (heat, acid, base, and oxidizing agent) on the stability of MPV, (2) to develop and optimize a univariate stability-indicating spectrophotometric assay for simultaneous quantification of MPV in the presence of its degradation products using a straightforward methodology (3) to study MPV degradation reactions' kinetics and (4) to assess the greenness of the proposed method to ensure its capacity of being a more accessible and safer option for quality control laboratories.

The one-step determination approach—using a simple direct spectrophotometric method—successfully analyzed MPV in the presence of its degradation products with no need for any treatment, calculation, or derivatization steps. Following the Green Analytical Chemistry (GAC) principles, the proposed method was developed considering the environmental impact to achieve a green analysis^19^. Three well-established green metrics, namely "National Environmental Method Index" (NEMI)^20^, Analytical Eco-Scale^21^, and "Green Analytical Procedure Index" (GAPI), were used as greenness profile checks^22^. The results showed a tripartite agreement on the greenness of the method without compromising the method's analytical performance. Owing to the direct analysis using a spectrophotometric technique, where the energy used is < 0.1 kWh per sample in addition to the green solvent used (water) and the minor consumption of solvent in method development and sample preparation compared to the chromatographic analysis, the method can be considered an excellent eco-friendly alternative.

## Experimental

### Instrumentation

Spectrophotometer: Shimadzu UV–Visible UV-1650 PC dual beam spectrophotometer (Kyoto/Japan), 1-cm quartz cells, bandwidth 1 nm, and scanning speed was 2800 nm/min with a 0.1 nm interval. Data acquisition was performed on UV-Probe 2.21 software.

LC–MS: Investigation of degradation products was performed using Agilent, Varian 1200L LC–MS system: Sample from the degradation solutions were withdrawn and eluted isocratically through an XSelect CSH C18 Column (130 Å, 5 µm, 4.6 mm × 250 mm) using acetonitrile: water (20:80 v/v) at 1.0 mL/min. The mass spectrometer operated in multiple reaction monitoring (MRM) with a positive ESI interface.

### Materials and reagents

Sodium hydroxide, hydrochloric acid, sodium chloride, and 30% Hydrogen peroxide H_2_O_2_ (Merck, Germany); deionized water was prepared by a water purification system (Purite, UK).

### Samples

Pure standard: Molnupiravir was kindly supplied by Eva pharma, Cairo-Egypt, and its purity was certified 99.79%. Degradation products: The degradation products were prepared using the procedure described below.

### Stock standard solutions

- Stock 5.0 × 10^–3^ M MPV standard solution in deionized water.
- Stock 5.0 M NaOH, 5.0 M HCl, and 5.0 M NaCl solutions in deionized water.

### Procedure

#### Study the effect of pH on the absorption spectra

The MPV stock standard solution (5 × 10^–3^ M) was diluted to prepare 2 × 10^–4^ M MPV working solutions in one of the following solvent solvents; deionized water, basic solution of 0.1 M NaOH, and acidic solution of 0.1 M HCl. The absorption spectra of each solution were recorded against its solvent within the wavelength range of 200–400 nm.

#### Forced degradation study of MPV

##### Forced acidic degradation

Into a 150 mL glass stoppered flask, transfer accurately 10.0 mL of MPV stock standard solution (5 × 10^–3^ M), add 50.0 mL of stock HCl solution (5.0 M), and reflux in a boiling water bath at 100 °C for 5 h, to accelerate the degradation process. Neutralize the solution with 50 mL of stock NaOH solution (5.0 M). The solution was quantitatively diluted to 250 mL with deionized water. The absorbance spectrum was recorded against 5.0 M NaCl solution within the 200–400 nm wavelength range. Mass spectrometry was utilized to investigate the degradation pattern.

##### Forced alkaline degradation

Into a 150 mL glass stoppered flask, transfer accurately 10.0 mL of MPV stock standard solution (5 × 10^–3^ M), add 50.0 mL of stock NaOH solution (5.0 M) to accelerate the degradation process, and reflux in a water bath at 100 °C for 5 h. Neutralize the solution with 50 mL of stock HCl solution (5.0 M). The solution was quantitatively diluted to 250 mL with deionized water. The absorbance spectrum was recorded against 5.0 M NaCl solution within the 200–400 nm wavelength range. Mass spectrometry was utilized to investigate the degradation pattern.

##### Forced oxidative degradation

Into a 150 mL glass stoppered flask, transfer accurately 10.0 mL of MPV stock standard solution (5 × 10^–3^ M), add 50.0 mL of stock H_2_O_2_ solution (30%) to accelerate the degradation process, reflux in a boiling water bath at 100 °C for 5 h, and then the flask was left open till dryness. The flask's content was reconstituted in deionized water and quantitatively diluted to 250 mL with deionized water. The absorbance spectrum was recorded against deionized water within the 200–400 nm wavelength range. Mass spectrometry was utilized to investigate the degradation pattern.

#### Construction of calibration curves

Different aliquots of MPV standard solution (5 × 10^–3^ M) were quantitatively diluted using deionized water to prepare 1.50 × 10^–5^–4.0 × 10^–4^ M MPV solutions. The absorbance at 270 nm was plotted against MPV concentration. We computed the regression equation to correlate the absorbance at 270 nm to MPV concentration.

#### Direct spectrophotometric determination of MPV in the presence of its degradation products

Laboratory prepared mixtures of MPV (1.5 × 10^–5^–4.0 × 10^–4^ M) and its degradation products (10%-90% degradation on molar basis). The absorbance of each solution at 270 nm was used to determine the concentration of intact MPV from its computed regression equation.

#### Molnupiravir degradation reactions' kinetics study

We refluxed MPV working standard solutions (2 × 10^–4^ M) in a boiling water bath at 100 °C in 0.1 M NaOH, 0.1 M HCl, and 30% H_2_O_2_ separately. We removed samples frequently and recorded their absorbance at 270 nm to determine the concentration of the intact MPV at each time interval using the regression equation. The results were used to plot each degradation reaction's zero, first, and second-order reaction kinetics.

## Results and discussion

**Figure 2:**
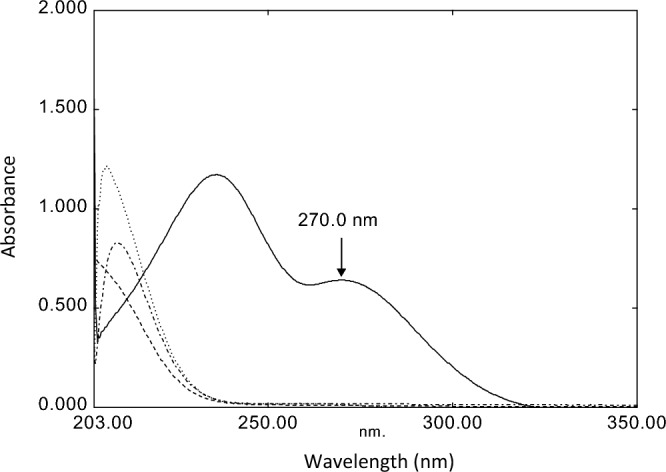
Absorption spectra of Molnupiravir (––), acid degradation product (– –), alkaline degradation product (---), Oxidation degradation product (– . –).

Molnupiravir is a new drug for COVID 19, structure shown in (Fig. 1). Being a prodrug with an ester functional group, it gets metabolized to NHC, which gets phosphorylated in the cells to form the pharmacologically active form^23^. It is essential to investigate MPV stability under stress conditions, how the drug molecule can be altered in thermal, acidic, basic, and oxidative conditions, and the different degradation kinetics. Moreover, in our research, we aimed to further invest in UV spectrophotometric technique to determine the MPV selectively in the presence of its degradation products by measuring MPV absorbance at 270 nm, where the degradation products show nil absorption, as shown in (Fig. 2).

**Figure 3:**
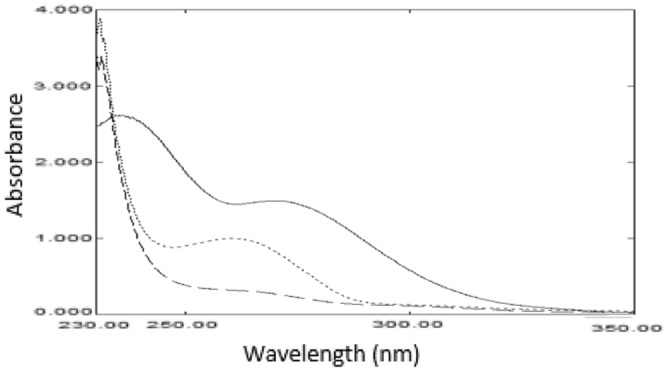
The absorption spectrum of 2 × 10^–4^ M molnupiravir solution (─) in deionized water, (---) aqueous 0.1 M NaOH, and aqueous 0.1 M HCl (─ ─).

We started by analyzing the impact of pH on the UV absorbance spectrum of MPV. We observed a significant difference in the absorbance spectrum in neutral, acidic, and alkaline media, (Fig. 3). To avoid false conclusions, the pH study aims to distinguish the shift in absorbencies caused by pH from that caused by degradation.

### Degradation process and proposed degradation pattern

To study the drug stability, MPV was subjected to various stress conditions that include thermal, acid, alkali, and oxidative degradation^24^.

#### Effect of heat

**Figure 4:**
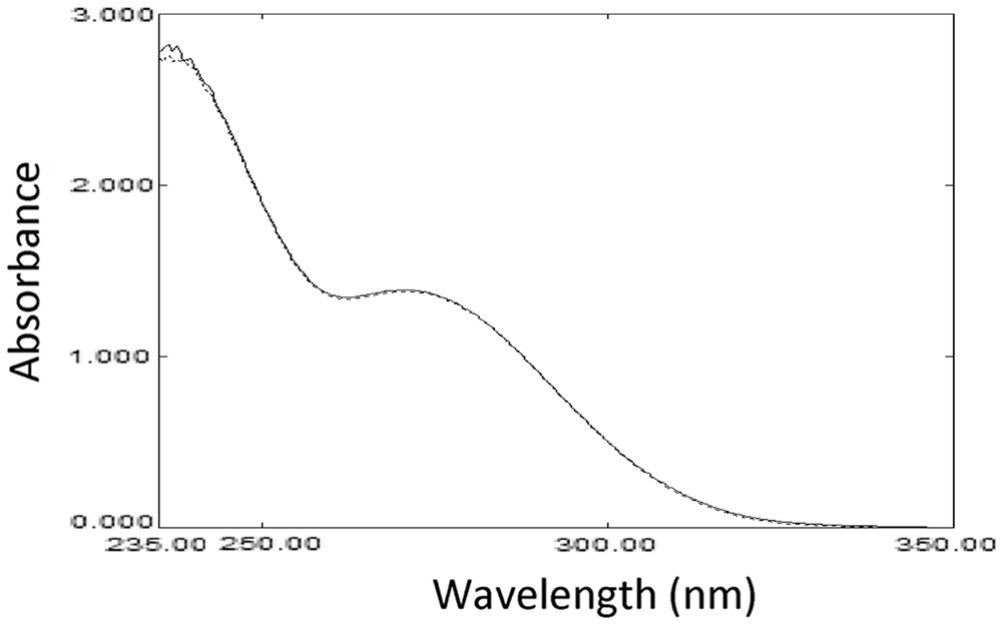
The absorption spectrum of aqueous 1 × 10^–4^ M Molnupiravir solution before heating (─) and after heating (---).

**Figure 5:**
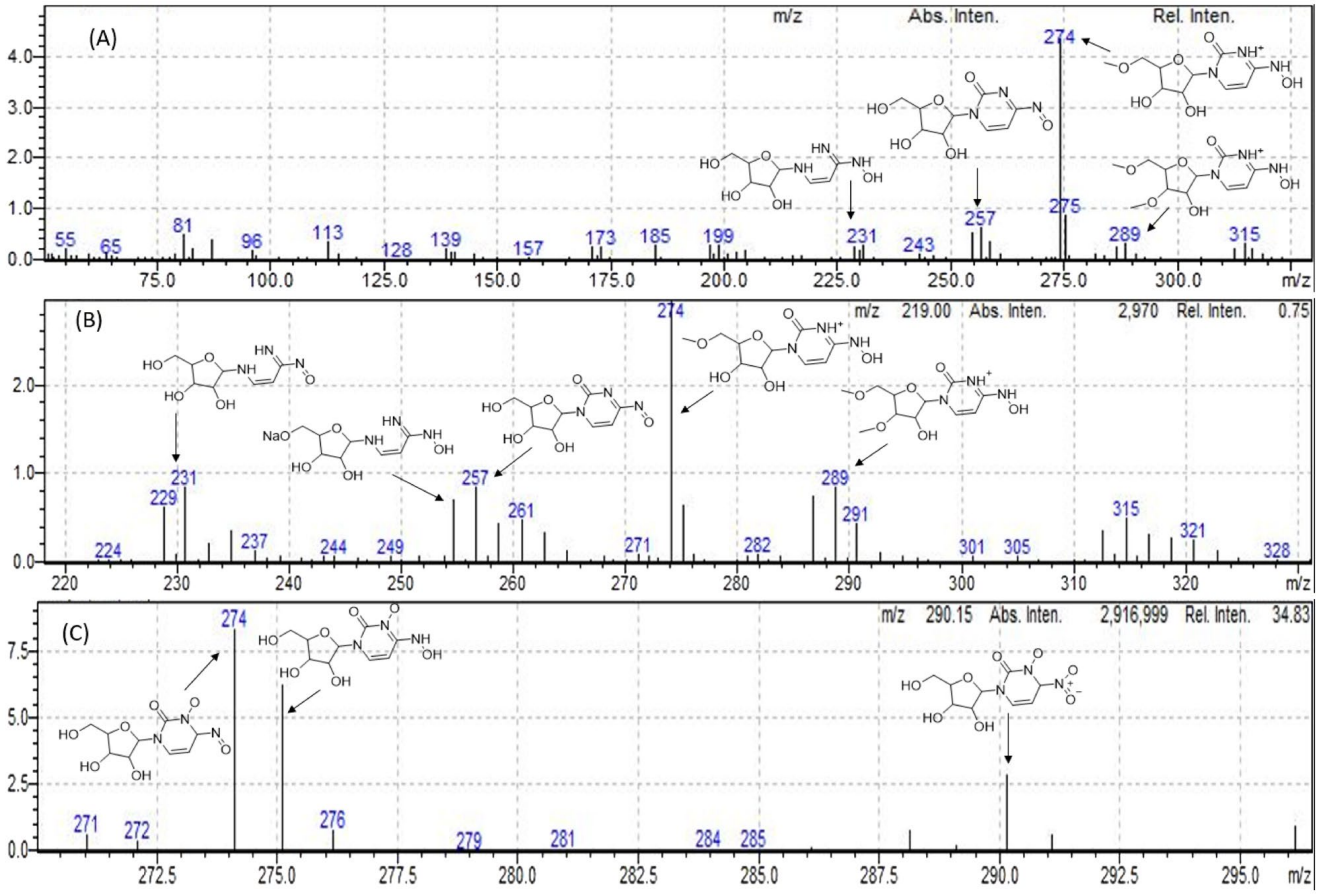
Mass spectra and the proposed degradation products (**A**) acid degradation, (**B**) base degradation, and (**C**) oxidative (30% H_2_O_2_) degradation.

The drug was stable upon exposure to thermal degradation via refluxing in a boiling water bath at 100 °C for 5 h. The UV absorbance spectrum appeared unaltered, and the mass spectra indicated no changes in the drug after and before dryness (Fig. 4) and (Fig. 5).

#### Effect of acid and base (hydrolytic degradation)

We performed MPV hydrolysis in acid and alkali solutions by refluxing it in a boiling water bath with 5.0 M HCl and 5.0 M NaOH for five hours, and the solutions were then neutralized with 5 M NaOH and 5 M HCl, respectively. Degradation was confirmed by comparing the absorption spectra, where the peak at 270 nm completely disappeared, as seen in (Fig. 2). The suggested degradation pattern was confirmed by mass spectrometry (Fig. 5A, B).

#### Effect of oxidizing agent

The oxidative degradation process was carried over by dissolving the drug in deionized water and then refluxing in a boiling water bath with H_2_O_2_ solution (30%). Reflux in a boiling water bath at 100 °C for 5 h. Then the flask was left open till dryness.

Degradation was confirmed by comparing the absorption spectra where the peak at 270 nm completely disappeared, as seen in Fig. 2. The suggested degradation pattern was confirmed by mass spectrometry (Fig. 5C).

### The direct spectrophotometric assay

The absorption spectra of MPV and its degradation products allowed a simple way to detect MPV in the presence of its degradation products as only MPV shows a clear absorbance peak at 270 nm while the degradants showed nil absorbance at the same wavelength. Consequently, a direct spectrophotometric method can be used for the drug analysis without any preliminary separation or derivatization steps (Fig. 2).

In the direct method, we construct calibration curves for the absorbance at 270 nm and MPV concentration to compute the regression equation (Table 1).

**Table 1:** Regression and validation data for the proposed stability-indicating spectrophotometric method.

Parameter	D^0^ Zero order
Slope^a^	6853.6
Intercept^a^	− 0.0091
Correlation coefficient^a^	0.9998
Linearity range (M)	1.50 × 10^–5^–4.0 × 10^–4^
Accuracy ± SD^b^	100.32 ± 1.62
Precision %RSD	
Intermediate^c^	1.899
Repeatability^c^	2.146
Quantification limit	1.50 × 10^–5^
Detection limit	0.48 × 10^–5^
Specificity	99.49 ± 1.63

^a^Average of three determinations.

^b^The accuracy (n = 3), average recovery of three Molnupiravir concentrations (1.0 × 10^–4^ M, 2.0 × 10^–4^ M, and 4.0 × 10^–5^ M).

^c^The repeatability and intermediate precision (n = 3), RSD% of three Molnupiravir concentrations (1.0 × 10^–4^ M, 2.0 × 10^–4^ M, and 4.0 × 10^–5^ M).

The ICH validation guidelines were followed^24^, as stated in Table 1. The method proved acceptable linearity, accuracy, and selectivity for MPV in presence of its hydrolytic and oxidative degradation products within a MPV concentration range of 1.50 × 10^–5^–4.0 × 10^–4^ M.

Linearity was assessed by analyzing six different concentrations of MPV in the range of 1.50 × 10^–5^–4.0 × 10^–4^ M. Calibration considered the practical linear range (Beer's law) to yield accurate and precise results, as shown in Table 1.

Accuracy was evaluated by applying the suggested method to determine different samples of MPV. The regression equation computed the MPV molar concentration. For precision testing, within-day repeatability was evaluated by analyzing three concentration levels of MPV. Likewise, the intermediate precision was studied on three consecutive days using three concentrations levels. Results showed an acceptable precision level, (Table 1).

The method specificity was confirmed at extremities by analyzing laboratory-prepared mixtures containing 10 to 90% degradation products, (Table 1).

### Statistical analysis

The study compared the results to those obtained by HPLC method reported by Reçber et al.^7^ We used the F-test to compare the precision (variance) and student’s t-test to compare the mean (accuracy) of the results. The two set of results did not significantly differ in either precision or accuracy within at 5% significance level, (Table 2).

**Table 2:** Statistical comparison between the results obtained using the proposed spectrophotometric method and the reported HPLC method for Molnupiravir assay^8^.

Parameter	D^0^ method	Reported method^a^
Mean	100.32	99.62
SD	1.624	0.795
n	6	6
Variance	2.637	0.632
Student's t-test^b^	0.972 (2.228)	
F-test^b^	4.173 (5.05)	

^a^HPLC method using a C18 column, water: acetonitrile (80:20) as mobile phase, and UV-detection at 240 nm.

^b^The values in parentheses are the corresponding theoretical values for t and F at P = 0.05.

### Kinetic study

**Figure 6:**
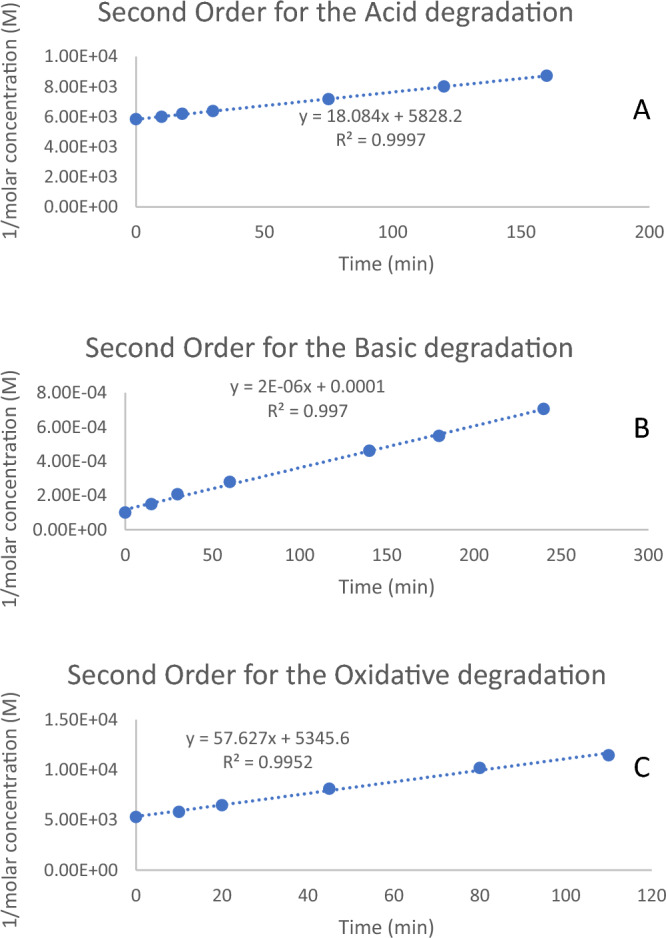
Second-order reaction kinetics for the degradation of Molnupiravir in (**A**) acidic, (**B**) basic, and (**C**) oxidative forced degradation conditions.

Thanks for the selectivity of the developed method, the study then applied the method to study the kinetics of the degradation reactions. The method selectively determined the concentration of the residual MPV at frequent time intervals from the inception of the degradation processes. The molar concentration of MPV was calculated directly from the absorbances at 270 nm using the regression equation. On the one hand, the zero-order ([MPV] versus time) and the first order (ln[MPV] versus time) plots expressed poor fitting for the three degradation reactions. The calculated correlation coefficients proved a proper fitting of the data to the second-order plots (1/[MPV] versus time) for the three degradation pathways (Fig. 6, Table 3). Thus, the second-order rate law governed the kinetics of the three degradation reactions. We calculated the rate constants and the half-life time of the three pathways. The alkaline degradation was the fastest, with a half-life of less than one hour under the mentioned experimental conditions (Fig. 6, Table 3).

**Table 3:** Second-order reaction kinetic parameters for Molnupiravir degradation.

	Acidic degradation	Basic degradation	Oxidative degradation
r	0.9998	0.9985	0.9976
k (${M}^{-1} {s}^{-1}$)	18.084	2 × 10^–6^	57.28
${t}_{1/2}$ (min)	322.28	50.00	93.32

### Greenness assessment

Green analytical chemistry is considered an integral part of sustainability^25^, and chemists are shifting to greener and more environmentally friendly methods^26^. We evaluated the method greenness profile using three different metrics: namely, NEMI^20^, Analytical Eco-Scale^21^, and GAPI tool^22^. The NEMI pictogram presents four quadrants [(1) persistent, bio-accumulative, and toxic (PBT), (2) hazardous, (3) corrosive, and (4) waste], for our method, the four quadrants are shaded green which clearly showed that the method passed the greenness check according to NEMI criteria, as seen in Table 4. To be ideally green, a method should score 100 in the Analytical Eco-Scale, our proposed method scored 94 after subtracting the penalty points. Accordingly, the suggested method is a perfectly green method, (Table 4). The GAPI tool was used for a more compressive assessment, and its analysis showed that our suggested method has minimal environmental impact, with eight green, five yellow, and two red fields. The three tools have a similar judgment on the greenness level of our method with a joint agreement on being a green method of analysis. A comparison with the reported methods using the same metrics showed that our suggested method is much greener with a higher Eco-scale value and more green quadrants in NEMI and more green fields in GABI. These findings may demonstrate the superiority of our proposed methodology.

**Table 4:** Comparing the Greenness profile for the proposed spectrophotometric method and the reported RP-HPLC ^8^ and LC–MS/MS^7^ methods.

Method/green metrics	Analytical ECO scale	Hazards and penalty points	NEMI	GABI
Proposed spectrophotometric method	Reagent			
	Water	0		
	Instruments			
	UV–Vis spectrophotometry	0		
	Occupational hazard	0		
	Waste	6		
	Total penalty points	∑ 6		
	Eco scale	94		
Reported RP-HPLC^a^	Reagent			
	Acetonitrile	8		
	Water	0		
	Instruments			
	HPLC	0		
	Occupational hazard	0		
	Waste	6		
	Total penalty points	∑ 14		
	Eco scale	86		
Reported LC–MS/MS^b^	Reagent			
	Acetonitrile	8		
	Ammonium acetate	3		
	Acetic acid	2		
	Instruments			
	LC–MS	1.5		
	Occupational hazard	0		
	Waste	6		
	Total penalty points	∑ 20.5		
	Eco scale	79.5		

^a^ HPLC method using a C18 column, water: acetonitrile (80:20) as mobile phase, and UV-detection at 240 nm.

^b^ LC–MS/MS method using a C18 column, with gradient elution of 1 mM Ammonium acetate in water (pH4.3) and 1 mM Ammonium acetate in acetonitrile.

## Concluding remarks

Molnupiravir is the first COVID 19 oral antiviral. Due to the global distribution of its users, it is essential to investigate its stability under a variety of stress conditions and to develop a simple stability indicating method for MPV. The drug is liable to hydrolytic (acidic and basic) and oxidative (30% H_2_O_2_) degradations and showed stability towards thermal conditions (100 °C for 5 h). The proposed spectrophotometric method presents a direct single-step procedure for the assay MPV with high accuracy and precision. In addition, the suggested method does not require any preliminary separation steps, various data manipulation, expensive instruments, or trained technicians. This adds potential advantages to our work, offering a cheap and accessible analytical method for use in moderately equipped analytical laboratories. The degradation pathways for hydrolytic and oxidative degradation were proposed, elucidated, and confirmed by mass spectrometry. The alkaline degradation showed the shortest half-life of less than one hour under the mentioned experimental conditions.

Furthermore, our proposed method hits very high scores/categories in different green matrix tools, being environment friendly with minimum hazardous effect on the environment and humans compared to the reported chromatographic ones. All the determination was done in deionized water (the greenest solvent), no derivatization needed, using minimum energy, and producing less waste. The proposed method can be the safer option for the routine analysis of Molnupiravir in the presence of its breakdown products in pure bulk powder and dosage form.

## Data Availability

All data are available from the corresponding author upon request.
